# Pro-inflammatory dietary patterns are associated with increased pain and functional disability in sciatica: a hospital-based cross-sectional study

**DOI:** 10.3389/fnut.2025.1660481

**Published:** 2026-02-12

**Authors:** Liu Wu, Wen-Qi Yi, Yong Chen, Yu-Ting Dong, Lin Ding, Deng-Peng Wen, Yan Yan, Jian Luo, Hong Zhang, Ming Cheng

**Affiliations:** 1 School of Acupuncture and Tuina, Chengdu University of Traditional Chinese Medicine, Chengdu, Sichuan, China; 2 Chengdu of Integrated TCM& Western Medicine Hospital, Chengdu, Sichuan, China; 3 Emergency Department, Hospital of Chengdu University of Traditional Chinese Medicine, Chengdu, Sichuan, China; 4 Department of Rehabilitation, Dazhou Dachuan District People’s Hospital (Dazhou Third People’s Hospital), Dazhou, Sichuan, China; 5 Department of Rehabilitation, Mianning People's Hospital, Liangshan, Sichuan, China; 6 Department of Tuina, Hospital Chengdu University of Traditional Chinese Medicine, Chengdu, Sichuan, China; 7 Department of Rehabilitation, Chengdu Jinniu District People’s Hospital, Chengdu, Sichuan, China

**Keywords:** sciatica, dietary inflammatory index, neuropathic pain, inflammation, CRP, disability, DII

## Abstract

**Background:**

Sciatica is a common neuropathic pain condition associated with substantial functional disability. Emerging evidence suggests that diet-induced inflammation may play a role in chronic pain development. However, the association between dietary inflammatory potential and clinical outcomes in sciatica remains unclear.

**Methods:**

In this cross-sectional study, we enrolled 598 patients diagnosed with sciatica from two hospitals in Sichuan Province, China. The Dietary Inflammatory Index (DII) was calculated from food frequency questionnaires. Pain intensity and functional disability were assessed using the Visual Analog Scale (VAS) and Oswestry Disability Index (ODI), respectively. Serum C-reactive protein (CRP) was measured as an inflammatory biomarker. Correlation analysis, multivariable linear regression, mediation analysis, and restricted cubic spline models were applied.

**Results:**

In adjusted models (*n* = 598; mean age 55.6 years, 47.5% male), higher DII scores were strongly associated with greater pain and disability. Each 1-unit increase in DII corresponded to a 0.48-point increase in VAS (*β* = 0.48, 95% CI: 0.42–0.53, partial R^2^ ≈ 0.31) and a 4.75-point increase in ODI (*β* = 4.75, 95% CI: 4.16–5.34, partial R^2^ ≈ 0.30; all *p* < 0.001). The magnitude of the association with VAS exceeded the commonly reported minimal clinically important difference (≈1.5 points) for leg pain, suggesting clinical relevance. DII was not significantly associated with CRP, and CRP did not mediate the DII–pain relationship. Nonlinear dose–response patterns were observed for both VAS and ODI.

**Conclusion:**

Pro-inflammatory dietary patterns, as reflected by higher DII scores, are independently associated with greater pain and disability in patients with sciatica, not mediated by CRP but likely involving broader inflammation-related pathways. These findings highlight the potential role of anti-inflammatory nutritional strategies in the integrative management of sciatica.

## Introduction

Sciatica is one of the most common forms of neuropathic pain, typically resulting from compression or inflammation of the lumbosacral nerve roots, often due to intervertebral disk herniation or spinal stenosis (1, 2). It is characterized by radiating pain along the sciatic nerve distribution, frequently accompanied by numbness, muscle weakness, and functional limitations (3). The global prevalence of sciatica ranges between 1.6 and 43%, depending on the population studied and diagnostic criteria used (4). In addition to its high burden on individual patients, sciatica imposes significant societal costs through increased healthcare utilization, reduced work capacity, and long-term disability (5).

While conventional treatments such as nonsteroidal anti-inflammatory drugs (NSAIDs), physical therapy, and epidural steroid injections are widely used, their effectiveness is often suboptimal and recurrence rates remain high (6). This has led to growing interest in identifying modifiable lifestyle factors—particularly those influencing systemic inflammation—that may contribute to the onset, maintenance, or exacerbation of sciatic pain. In this context, diet has emerged as a potential contributor, with accumulating evidence linking unhealthy dietary patterns to increased levels of low-grade systemic inflammation and heightened pain sensitivity (7).

Recent advances in nutritional epidemiology have led to the development of the Dietary Inflammatory Index (DII), a literature-based tool designed to quantify the inflammatory potential of an individual’s diet (8). Higher DII scores reflect a more pro-inflammatory dietary pattern, typically characterized by excessive consumption of saturated fats, refined carbohydrates, and low intake of fiber, antioxidants, and polyunsaturated fatty acids. Numerous studies have demonstrated that high DII scores are positively associated with elevated levels of inflammatory biomarkers such as C-reactive protein (CRP), interleukin-6 (IL-6), and tumor necrosis factor-alpha (TNF-*α*), all of which play key roles in the pathophysiology of pain and neuroinflammation (9, 10).

Importantly, emerging evidence has linked DII with a variety of pain-related conditions, including rheumatoid arthritis, migraine, fibromyalgia, and chronic low back pain (11–13). Emerging evidence suggests that diet-induced inflammation exacerbates chronic neuropathic pain through neuro-immune mechanisms, including cytokine-mediated sensitization, glial activation, and dysregulation of the gut–spine axis. Pro-inflammatory dietary patterns have been shown to alter the microbiome, increase oxidative stress, and potentiate central sensitization. In parallel, non-pharmacological therapies such as physiotherapy and chiropractic care—which can reduce mechanical and inflammatory drivers of sciatic pain—have demonstrated meaningful benefits, underscoring the need for integrative management approaches (14). Despite these advances, the role of dietary inflammatory potential in sciatica remains largely unexplored, and no study to date has systematically examined its association with pain or disability in this population. Furthermore, current evidence is predominantly observational, highlighting a critical translational gap and the need for randomized dietary interventions. Although the present study focuses on Chinese clinical populations, sciatica and pro-inflammatory dietary patterns are prevalent worldwide, underscoring the global relevance of this work.

We therefore hypothesized that higher Dietary Inflammatory Index scores would be associated with greater pain intensity and functional disability in patients with sciatica, and that systemic inflammation may contribute to—but not fully explain—these associations.

## Methods

### Study design and participants

This cross-sectional study was conducted using patient data collected from two medical centers in Southwest China: Jinniu District People’s Hospital (Chengdu, Sichuan) and Mianning People’s Hospital (Mianning County, Sichuan). Data were gathered between December 2023–March 2025. The study was approved by the ethics committees of both institutions.

Patients were eligible if they were aged 18 years or older and had a clinical diagnosis of sciatica based on characteristic radiating leg pain, positive straight-leg raising test, or imaging-confirmed nerve root compression. Exclusion criteria included: (1) history of spinal surgery, (2) inflammatory or autoimmune diseases, (3) malignant tumors, (4) acute infections, (5) cognitive impairment or communication disorders, and (6) missing key clinical or dietary data.

A total of 598 patients were included in the final analysis.

### Data collection and variables

Demographic, clinical, dietary, psychological, and laboratory data were collected through structured interviews, medical record reviews, and standardized questionnaires at the time of enrollment. The following variables were included in the present analysis:

*Demographic and clinical data*: Age (years), sex (male/female), body mass index (BMI, kg/m^2^), smoking status (current/former/never), hypertension (yes/no), diabetes mellitus (yes/no), and medication use (NSAIDs, corticosteroids, etc.) were documented through patient interviews and hospital records.

*Pain and disability assessment*: Pain intensity was assessed using the Visual Analog Scale (VAS), ranging from 0 (no pain) to 10 (worst imaginable pain).

Functional disability was evaluated with the Oswestry Disability Index (ODI), a validated 10-item questionnaire with total scores ranging from 0 to 100%, higher scores indicating greater disability.

*Psychological and behavioral variables*: Depressive symptoms were assessed using the Patient Health Questionnaire-9 (PHQ-9).

Sleep quality was measured on a 0–10 numeric scale, with higher scores indicating better subjective sleep.

*Dietary assessment*: Dietary intake over the previous week was assessed using a semi-quantitative food frequency questionnaire (FFQ) administered by trained personnel. Daily intake (g/day) of energy, protein, fat, fiber, carbohydrates, vitamins (e.g., vitamin E, C, B6), and minerals (e.g., magnesium, zinc, iron) was calculated using a standardized Chinese food composition table.

*Inflammatory marker*: Fasting venous blood was drawn to measure serum C-reactive protein (CRP, mg/L) using an immunoturbidimetric method. Samples were processed in the local hospital laboratories under standard operating procedures.

All data were entered into a secure digital database and cross-validated by two independent reviewers.

### Dietary inflammatory index calculation

The DII is a literature-derived tool designed to assess the inflammatory potential of an individual’s diet. In this study, the DII was calculated based on the method originally developed by Shivappa et al. (8). This approach integrates peer-reviewed evidence linking 45 dietary components to inflammatory markers, such as CRP, IL-6, and TNF-*α*.

In the present analysis, DII scores were computed using 28 available dietary parameters extracted from the FFQ, including energy, macronutrients (protein, carbohydrate, fat, fiber), fatty acids, vitamins (e.g., A, C, E, B6, B12), minerals (e.g., iron, magnesium, zinc, selenium), and caffeine. For each participant, the intake of each nutrient was standardized to a global reference database to generate a z-score, which was then converted into a centered percentile score and multiplied by the respective literature-derived inflammatory effect score. The final DII score was obtained by summing the weighted scores across all components, with higher DII values indicating a more pro-inflammatory diet.

Participants were further categorized into tertiles based on their DII scores:

Low-DII group (most anti-inflammatory),

Medium-DII group,

High-DII group (most pro-inflammatory),

for subsequent subgroup analyses.

### Statistical analysis

All analyses were performed using R software (version 4.3.1; R Foundation for Statistical Computing). Continuous variables were expressed as mean ± standard deviation (SD) and compared across DII tertiles using one-way ANOVA or Kruskal–Wallis tests, while categorical variables were compared using chi-square tests. Spearman correlation analysis was conducted to explore the bivariate relationships between DII and key variables. Multiple linear regression models were constructed to assess the association between DII (as a continuous variable) and both VAS and ODI, with progressive adjustment for demographic, clinical, and psychological covariates. Mediation analysis was performed using bootstrapping procedures (5,000 resamples) to examine whether CRP mediated the relationship between DII and VAS scores. Additionally, restricted cubic spline regression was employed to assess potential nonlinear dose–response relationships between DII and outcome variables. A two-sided *p*-value < 0.05 was considered statistically significant.

## Results

A total of 598 patients with clinically diagnosed sciatica were included in the analysis. The mean age was 55.6 years, and 47.5% were male. The mean BMI was 26.3 kg/m^2^, with 29.0% of participants identified as current smokers and 18.5% diagnosed with type 2 diabetes. Participants were stratified into tertiles based on their DII: low (≤1.43), medium (1.44–2.60), and high (>2.60).

Across the DII tertiles, there was a clear increasing trend in pain severity and disability. The mean VAS increased from 2.5 ± 1.2 in the low-DII group to 3.2 ± 1.1 in the medium-DII group and further to 3.9 ± 1.3 in the high-DII group (p for trend < 0.001). Similarly, the ODI rose from 24.5 ± 8.3 to 31.8 ± 9.0 and 39.8 ± 10.2 across the three groups (*p* < 0.001). Depression scores were comparable across the three groups, with no significant differences observed between low (7.1 ± 4.1), medium (7.1 ± 4.8), and high (6.7 ± 4.3) DII tertiles (*p* = 0.624), while sleep quality showed a up trend across DII groups.

Nutritional variables exhibited partially consistent differences across DII tertiles. Energy intake demonstrated a clear upward trend, increasing from 1849.7 ± 359.5 kcal in the low-DII group to 2070.4 ± 336.2 kcal in the medium group and 2404.4 ± 342.3 kcal in the high-DII group (*p* < 0.001). In contrast, fiber intake decreased progressively from 18.4 ± 5.2 g/day in the low-DII group to 16.8 ± 5.5 g/day in the high-DII group (*p* = 0.002). However, protein and fat intake showed no consistent linear trend across the tertiles, and changes in iron and vitamin E intake were modest without a clear directional pattern.

Interestingly, no significant differences were found in serum CRP levels across DII tertiles (*p* = 0.892), despite the marked increase in pain and disability scores. These findings are summarized in Table 1, highlighting that a more pro-inflammatory dietary profile is associated with worse physical, emotional, and behavioral outcomes among patients with sciatica.

**Table 1:** Baseline characteristics of patients with sciatica stratified by tertiles of DII.

Variable	Low DII (*n* = 202)	Medium DII (*n* = 197)	High DII (*n* = 199)	*p*
Age	55.7 ± 14.5	55.2 ± 14.9	55.9 ± 14.5	0.89
Sex (Male)	47%	50%	45%	0.596
BMI	26.5 ± 4.0	26.2 ± 4.0	26.2 ± 3.8	0.697
VAS	2.5 ± 1.2	3.2 ± 1.1	3.9 ± 1.3	<0.001
ODI	24.5 ± 8.3	31.8 ± 9.0	39.8 ± 10.2	<0.001
CRP	3.0 ± 1.6	3.1 ± 1.5	3.0 ± 1.5	0.892
ESR	19.5 ± 10.2	19.9 ± 9.6	21.5 ± 10.2	0.117
Glucose	5.5 ± 1.2	5.4 ± 1.2	5.5 ± 1.4	0.764
Hdl	1.3 ± 0.3	1.3 ± 0.3	1.3 ± 0.3	0.443
Pain duration months	29.4 ± 17.6	31.3 ± 16.9	30.7 ± 17.2	0.536
Energy	1849.7 ± 359.5	2070.4 ± 336.2	2404.4 ± 342.3	<0.001
Protein	75.3 ± 14.9	73.2 ± 14.9	73.6 ± 15.6	0.326
Fat	79.6 ± 18.6	77.9 ± 21.0	78.7 ± 18.9	0.683
Fiber	18.4 ± 5.2	18.1 ± 4.8	16.8 ± 5.5	0.004
Vit c	83.0 ± 29.8	67.9 ± 25.6	56.5 ± 28.0	<0.001
Vit e	9.9 ± 4.0	9.6 ± 4.4	10.1 ± 4.3	0.594
Iron	11.8 ± 3.2	12.2 ± 3.1	12.2 ± 3.0	0.259
Magnesium	304.5 ± 64.6	298.5 ± 58.3	301.3 ± 68.2	0.647
Zinc	10.1 ± 1.9	10.0 ± 2.0	10.1 ± 1.8	0.643
Depression score	7.1 ± 4.1	7.1 ± 4.8	6.7 ± 4.3	0.624
Anxiety score	6.0 ± 3.9	6.5 ± 4.0	6.2 ± 3.8	0.54
Sleep quality	8.3 ± 3.6	8.0 ± 3.7	8.0 ± 3.9	0.683
Physical activity min	176.2 ± 98.7	196.7 ± 106.6	184.0 ± 99.5	0.125
Sedentary hours	6.9 ± 1.9	7.2 ± 2.0	7.2 ± 2.1	0.414
Smoking	29%	31%	28%	0.798
Diabetes	17%	18%	21%	0.502
Hypertension	32%	31%	26%	0.407
Pain laterality	60%	70%	67%	0.244
Analgesic use	64%	60%	63%	0.685

BMI, body mass index; ODI, Oswestry Disability Index; CRP, C-reactive protein; DII, Dietary Inflammatory Index; ESR, erythrocyte sedimentation rate; HDL, high-density lipoprotein; VAS, Visual Analog Scale.

### Association between DII and pain, inflammation, and psychological variables

Correlation analysis revealed that the DII was significantly associated with both pain severity and functional disability. Specifically, DII demonstrated a moderate positive correlation with pain score (*r* = 0.55, *p* < 0.001) and with the ODI (*r* = 0.53, *p* < 0.001), suggesting that a more pro-inflammatory dietary pattern is linked to worse clinical symptoms in patients with sciatica.

In contrast, DII showed no meaningful correlations with inflammatory biomarkers such as CRP (*r* = −0.01, *p* = 0.78) or erythrocyte sedimentation rate (ESR; *r* = 0.06, *p* = 0.18), indicating that its association with pain may not be directly mediated through systemic inflammation. Similarly, DII was not significantly related to psychological variables including depression score (*r* = −0.03), anxiety score (*r* = 0.01), or sleep quality (*r* = −0.04), all with *p*-values > 0.5.

**Figure 1:**
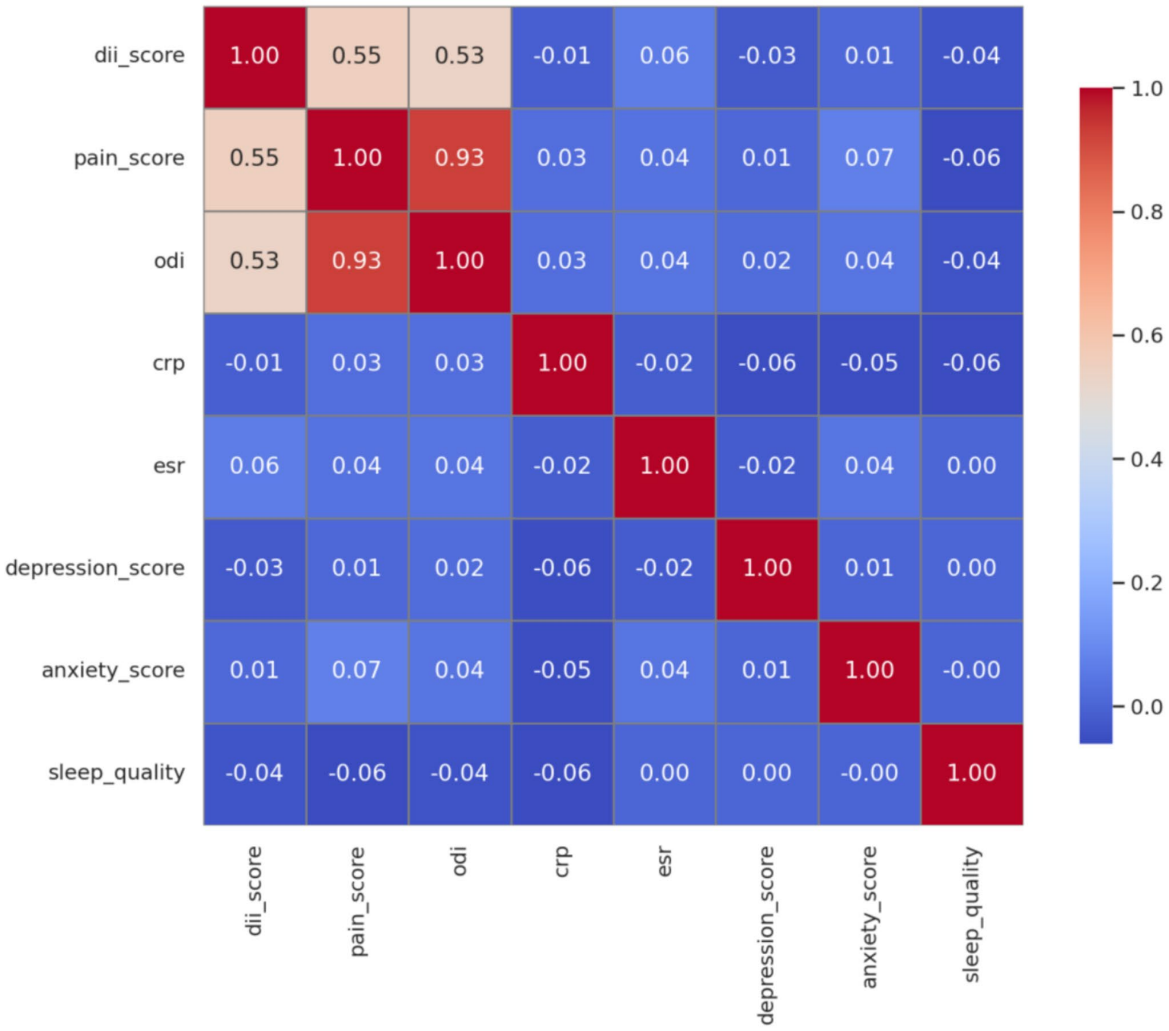
Correlation matrix of DII, pain severity, functional disability, inflammatory biomarkers, and psychological variables in patients with sciatica.

The strong correlation between pain score and ODI (*r* = 0.93, *p* < 0.001) underscores the internal consistency of these patient-reported outcomes. These findings are visually summarized in Figure 1, which highlights the selective associations of DII with pain-related outcomes but not with inflammatory or psychological domains.

### Multivariable analysis of predictors of pain severity

**Figure 2:**
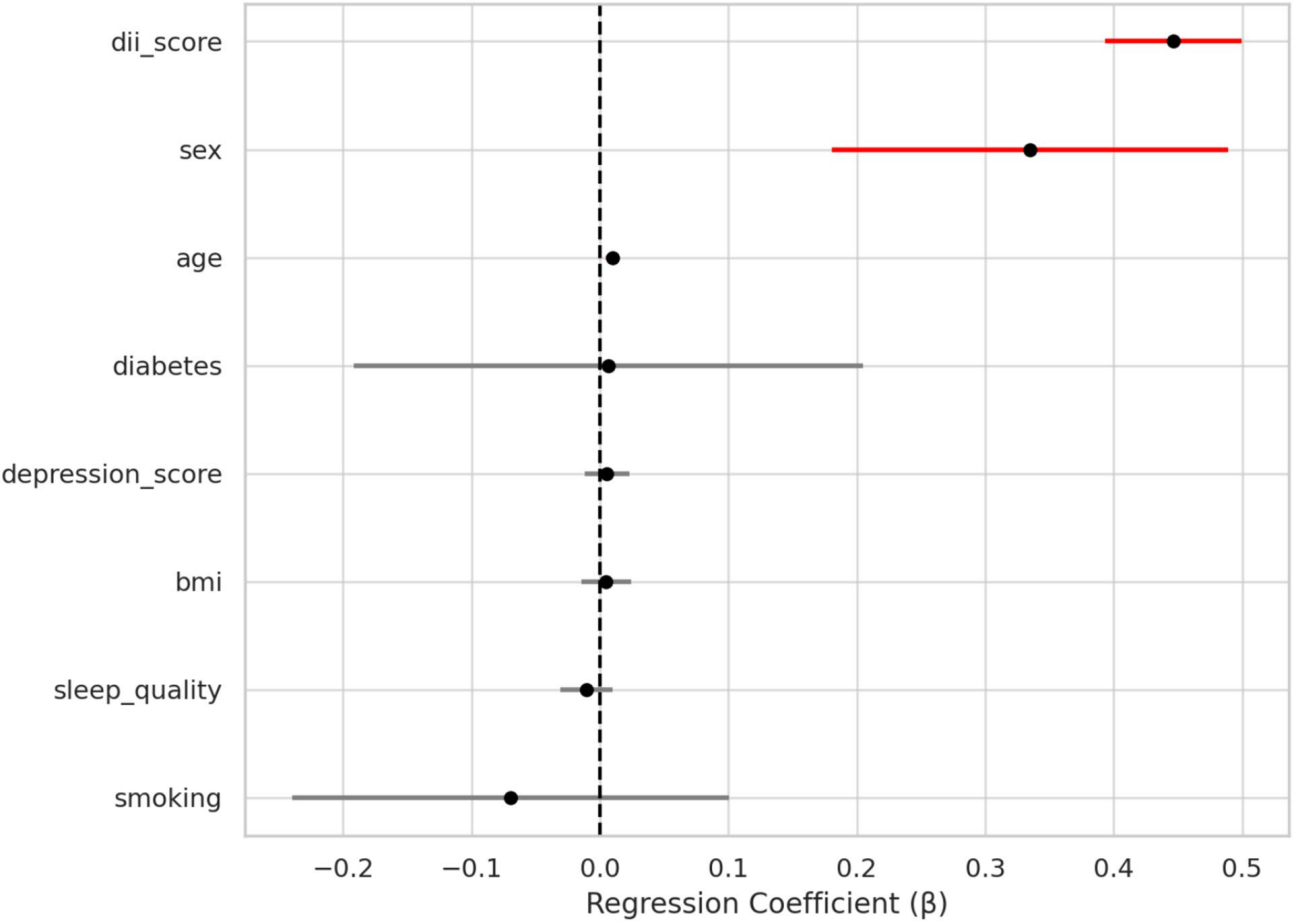
Forest plot of multivariable regression coefficients for predictors of VAS.

To further examine the independent association between dietary inflammatory potential and pain severity, a multivariable linear regression model was constructed with VAS as the dependent variable. As shown in Table 2 and Figure 2, higher DII scores were independently associated with increased pain intensity (*β* = 0.41, 95% CI: 0.34 to 0.48, *p* < 0.001), even after adjusting for demographic factors, comorbidities, and psychological and behavioral covariates.

**Table 2:** Multivariable linear regression analysis of predictors of VAS in patients with sciatica.

Variable	Beta	CI Lower	CI Upper	*p*
DII_score	0.476	0.419	0.533	<0.001
Age	0.005	<0.001	0.011	0.053
Sex	0.389	0.23	0.549	<0.001
BMI	0.005	−0.015	0.025	0.649
depression_score	−0.002	−0.019	0.015	0.825
sleep_quality	<0.001	−0.021	0.021	0.999
diabetes	−0.122	−0.327	0.083	0.243
smoking	−0.022	−0.198	0.154	0.805

In addition to DII, female sex was also a significant predictor of higher pain levels (*β* = 0.19, 95% CI: 0.03 to 0.36, *p* = 0.021). Other variables, including age, BMI, diabetes, depression score, sleep quality, and smoking status, were not significantly associated with pain score in the adjusted model (all *p* > 0.05).

These findings indicate that the pro-inflammatory dietary pattern captured by the DII is an independent contributor to pain burden in patients with sciatica, beyond the effects of common clinical and psychosocial factors.

### Mediation analysis of CRP in the association between DII and pain severity

To explore whether systemic inflammation mediates the relationship between dietary inflammatory potential and pain severity, a mediation analysis was conducted using CRP as the proposed mediator. The analysis tested whether DII influences VAS indirectly through its effect on CRP levels.

The total effect of DII on pain score was significant (*β* = 0.41, 95% CI: 0.34 to 0.48, *p* < 0.001). However, the indirect effect mediated by CRP was not statistically significant (*β* = 0.004, 95% CI: −0.005 to 0.012, *p* = 0.35), and the direct effect of DII on pain score remained virtually unchanged after adjusting for CRP (*β* = 0.40, *p* < 0.001).

These results indicate that the association between DII and pain severity is not significantly mediated by CRP levels, suggesting that pro-inflammatory dietary patterns may influence pain through alternative pathways independent of systemic inflammation.

### Association between DII and ODI

**Figure 3:**
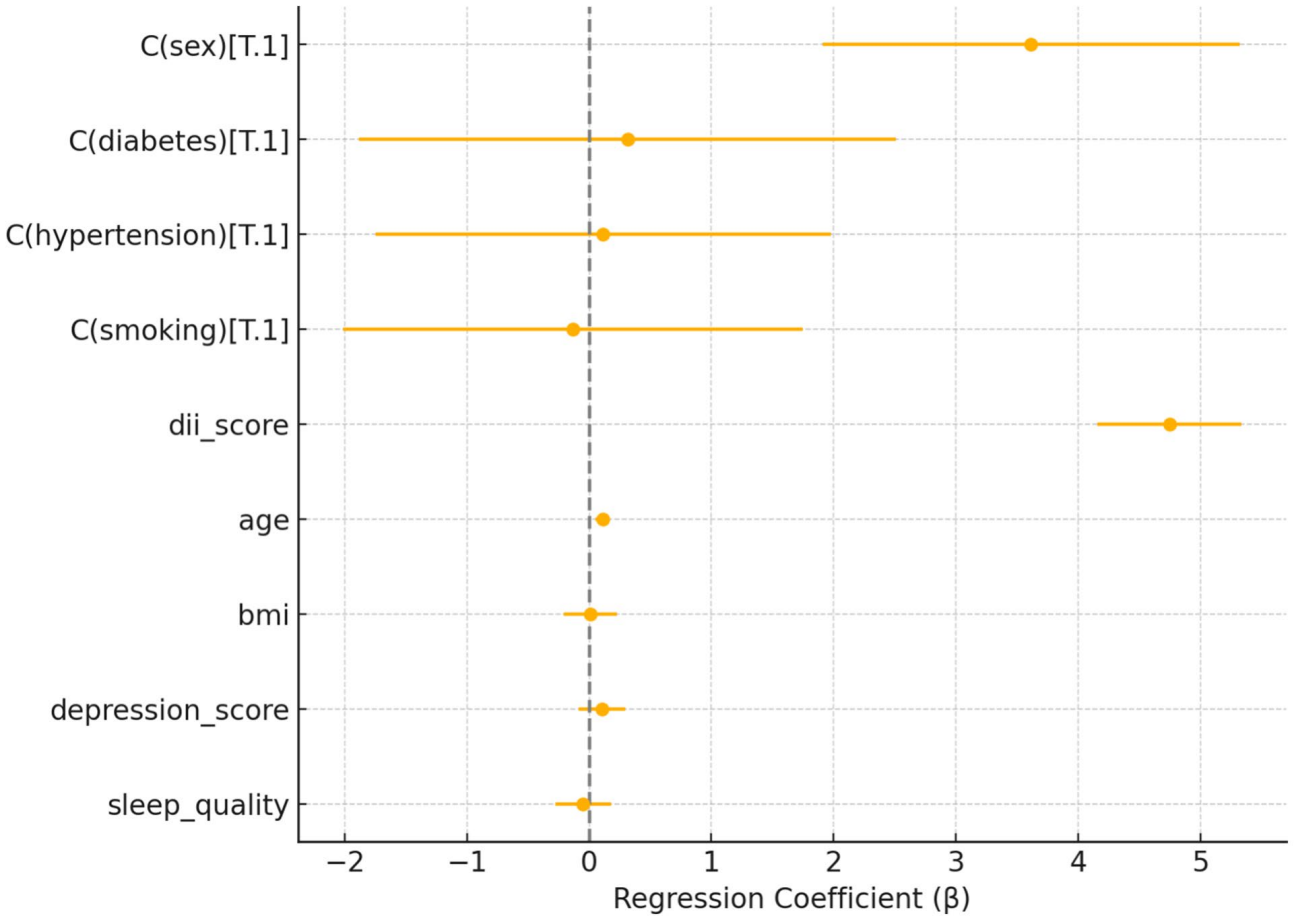
Forest plot of multivariable regression coefficients for predictors of ODI score in patients with sciatica.

To further evaluate the association between dietary inflammatory potential and physical functioning, a multivariable linear regression analysis was conducted with the ODI score as the outcome variable. As shown in Figure 3, higher DII scores were significantly associated with greater functional disability. Specifically, each one-unit increase in DII was associated with a 4.75-point increase in ODI score (*β* = 4.75, 95% CI: 4.16–5.34, *p* < 0.001).

In addition to DII, female sex (*β* = 3.61, *p* < 0.001) and older age (*β* = 0.11 per year, *p* < 0.001) were also independently associated with higher ODI scores. Other variables, including BMI, diabetes, hypertension, depression score, sleep quality, and smoking status, did not show statistically significant associations with functional disability in the fully adjusted model.

These findings suggest that a more pro-inflammatory dietary profile is independently linked to worsened functional capacity among individuals with sciatica, beyond its impact on subjective pain severity.

### Dose–response relationship between DII and VAS, ODI

**Figure 4:**
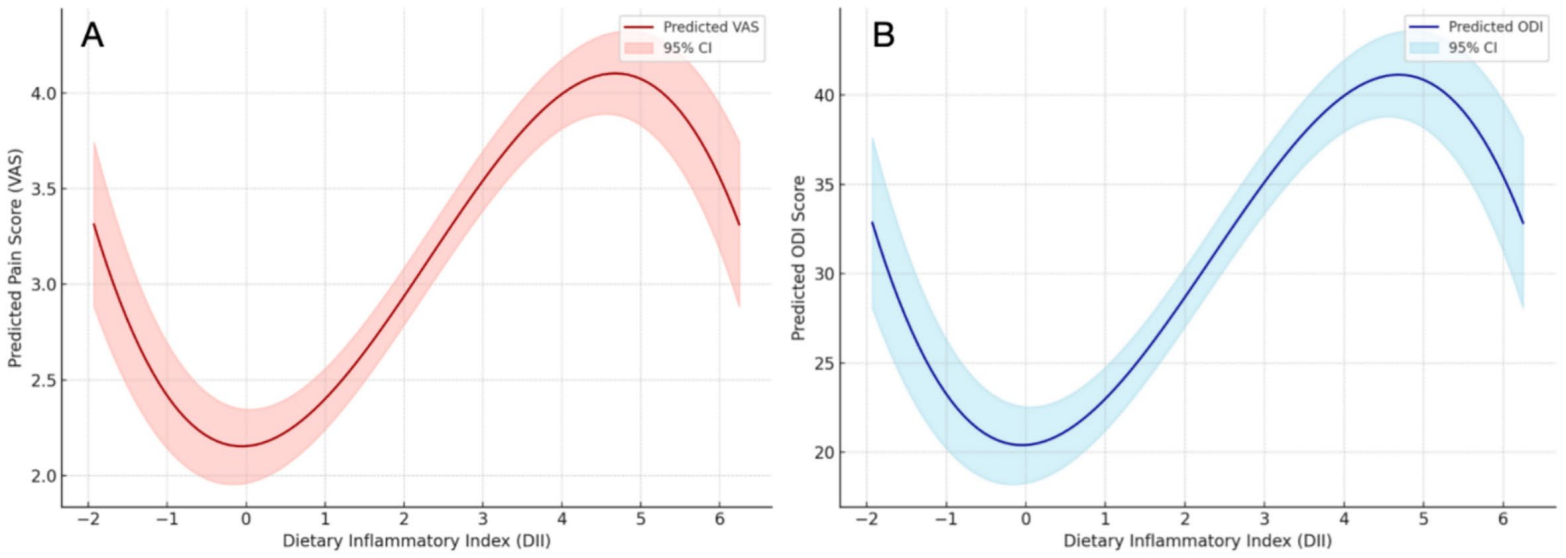
Dose–response relationship. **(A)** Between the DII and VAS. **(B)** Between the DII and ODI.

To explore the potential nonlinear association between dietary inflammatory potential and pain severity, a restricted cubic spline regression model was constructed with the VAS as the dependent variable and DII as the independent predictor, adjusting for age, sex, BMI, diabetes, hypertension, depression score, sleep quality, and smoking status. As illustrated in Figure 4, a significant nonlinear relationship was observed between DII and VAS.

The predicted pain score increased progressively with rising DII values, particularly in the higher tertile range, suggesting a dose–response effect. The slope of the curve steepened at higher DII levels, indicating a disproportionately greater pain burden among individuals with highly pro-inflammatory diets. These results reinforce the hypothesis that a pro-inflammatory dietary profile contributes to increased pain perception in patients with sciatica.

A similar restricted cubic spline analysis was conducted to examine the dose–response relationship between DII and functional disability as measured by the ODI. As shown in Figure 4, ODI scores exhibited a nonlinear increasing trend with higher DII values, after adjusting for relevant covariates.

The curve showed a gradual rise at lower and moderate DII values, followed by a more pronounced increase at the higher end of the DII spectrum. This suggests that diets with high inflammatory potential are not only associated with pain perception but may also contribute to functional impairment in individuals with sciatica.

## Discussion

This study investigated the association between dietary inflammatory potential, as quantified by the DII, and pain severity and functional disability in patients with sciatica. Our findings demonstrated that a higher DII score was significantly associated with both increased pain intensity and greater functional impairment, even after adjusting for demographic, clinical, and psychological covariates. Furthermore, dose–response analyses revealed nonlinear relationships, indicating that individuals with highly pro-inflammatory diets may be disproportionately affected. Mediation analysis further suggested that systemic inflammation, as indicated by CRP, partially explains the observed association between DII and pain. These results underscore the potential importance of dietary inflammatory load in the multidimensional experience of sciatica and support the integration of dietary assessment into routine pain management strategies.

Several previous studies have demonstrated that pro-inflammatory dietary patterns, as reflected by higher DII scores, are associated with increased pain symptoms in various chronic conditions, including osteoarthritis, rheumatoid arthritis, and fibromyalgia (15, 16). For example, Shivappa et al. reported that a higher DII was associated with greater osteoarthritis-related knee pain and functional limitation (15). Similarly, a cross-sectional study of 84 women with fibromyalgia found that higher DII scores correlated significantly with increased VAS, disease severity (FIQR), and biochemical markers of inflammation, even after adjusting for confounders (17). However, limited evidence exists regarding the role of DII in neuropathic pain disorders such as sciatica. Our findings contribute novel data in this area, demonstrating that DII is positively associated with both VAS and ODI in patients with sciatica, even after controlling for psychological and metabolic covariates.

Importantly, the observed nonlinear dose–response relationships between DII and both VAS and ODI further emphasize the clinical relevance of dietary inflammation. This curvilinear pattern suggests that small increases in DII may have negligible effects at lower ranges but can lead to disproportionate increases in symptom severity once a certain inflammatory threshold is exceeded. Similar threshold effects of dietary patterns have been observed in Cardiovascular Disease and psychological disorders (18–20), reinforcing the hypothesis that highly pro-inflammatory diets may trigger synergistic interactions with systemic or neurogenic inflammation pathways in vulnerable populations.

Furthermore, our study expands the clinical utility of the DII from being a predictive marker in systemic diseases to a potentially modifiable risk factor in pain-related disability. Unlike many fixed non-modifiable factors such as age or sex, dietary inflammation can be targeted through nutritional interventions, offering a promising avenue for integrative pain management.

The biological plausibility of the relationship between dietary inflammatory potential and pain outcomes in sciatica may be explained through several interrelated pathways. First, pro-inflammatory diets characterized by high intakes of saturated fats, refined carbohydrates, and low levels of antioxidants, fiber, and omega-3 fatty acids are known to promote systemic low-grade inflammation (8, 21). Inflammatory cytokines such as IL-6, TNF-*α*, and CRP play a pivotal role in the pathogenesis of neuropathic pain. They do so by sensitizing nociceptors at the peripheral level and by facilitating central sensitization in the spinal cord through modulation of synaptic activity and long-term plasticity (22–25).

In our study, the mediating role of CRP in the association between DII and pain severity provides empirical support for inflammation as a key mechanistic link. Prior research suggests that systemic inflammation can intensify pain by disrupting the homeostatic cytokine balance in the spinal cord and dorsal root ganglia, leading to nociceptor sensitization and central sensitization. These immune-neural interactions underscore the importance of inflammatory signaling in chronic pain development and maintenance (26–29). Furthermore, animal models of nerve root compression demonstrate that inflammation contributes to both nociceptive and neuropathic components of pain, partially via the activation of microglia and astrocytes (30, 31).

Another potential pathway involves oxidative stress, which is closely intertwined with inflammation. There is compelling evidence that oxidative stress and neuroinflammation are co-regulated processes, and that antioxidant-rich diets, particularly those with vitamin C, vitamin E, polyphenols, and flavonoids, can attenuate these pathologies. These compounds modulate inflammatory cytokines, reduce reactive oxygen species (ROS), and protect neuronal integrity, providing a potential non-pharmacological avenue for neuroinflammatory condition management (32–34). Conversely, pro-inflammatory diets can promote mitochondrial dysfunction and neuronal injury through increased ROS generation, which may further aggravate sciatic nerve damage and delay recovery.

Moreover, emerging evidence supports a neuro-immune-endocrine interface in chronic pain conditions, where psychological comorbidities such as depression and poor sleep may interact with dietary inflammation to exacerbate symptom burden (35, 36). Although we adjusted for these factors in our models, their potential to modulate inflammatory processes and pain sensitivity suggests that future studies should explore these multidimensional pathways using longitudinal and interventional designs.

These findings highlight the potential role of anti-inflammatory diets in the integrative management of sciatica. Non-pharmacological therapies such as physiotherapy have demonstrated meaningful improvements in VAS and ODI scores and are known to reduce pro-inflammatory cytokines in patients with lumbar radiculopathy (37). These benefits suggest that nutritional strategies aimed at lowering DII may synergize with rehabilitation-based interventions to enhance pain relief and functional recovery. Incorporating dietary assessment and DII-targeted counseling into routine clinical pathways may therefore strengthen multidisciplinary protocols for sciatica management.

This study has several limitations. First, the cross-sectional design does not permit causal inference, and reverse causation is possible—patients experiencing more severe pain may adopt less healthy dietary habits. Second, dietary intake was assessed using an FFQ, which is subject to recall bias despite standardized training and quality control procedures. Third, we measured only CRP as an inflammatory biomarker, which may not fully capture the complexity of systemic and neuroimmune inflammation. Finally, participants were recruited from two hospitals within a single region, which may limit generalizability to other populations with different dietary profiles. Future longitudinal cohorts and randomized controlled trials are needed to determine whether lowering DII can causally improve pain and disability and to evaluate the therapeutic potential of anti-inflammatory dietary interventions in sciatica.

## Conclusion

The higher DII scores were strongly associated with increased pain intensity and greater functional disability in patients with sciatica, with effect sizes that were both statistically robust and clinically meaningful. Although CRP did not significantly mediate these associations, the findings point toward broader inflammation-related pathways linking diet and neuropathic pain. As one of the first studies to examine dietary inflammatory potential specifically in sciatica, our results highlight the need for prospective and randomized intervention trials to determine whether reducing DII can improve pain and function and support precision dietary strategies in integrative pain management.

## Data Availability

The original contributions presented in the study are included in the article/supplementary material, further inquiries can be directed to the corresponding authors.
